# Estimating the Independent Effects of Multiple Pollutants in the Presence of Measurement Error: An Application of a Measurement-Error–Resistant Technique

**DOI:** 10.1289/ehp.7286

**Published:** 2004-09-07

**Authors:** Ariana Zeka, Joel Schwartz

**Affiliations:** Exposure, Epidemiology, and Risk Program, Environmental Health Department, Harvard School of Public Health, Boston, Massachusetts, USA

**Keywords:** air pollution, carbon monoxide, daily mortality, measurement error, particulate matter

## Abstract

Misclassification of exposure usually leads to biased estimates of exposure–response associations. This is particularly an issue in cases with multiple correlated exposures, where the direction of bias is uncertain. It is necessary to address this problem when considering associations with important public health implications such as the one between mortality and air pollution, because biased exposure effects can result in biased risk assessments. The National Morbidity and Mortality Air Pollution Study (NMMAPS) recently reported results from an assessment of multiple pollutants and daily mortality in 90 U.S. cities. That study assessed the independent associations of the selected pollutants with daily mortality in two-pollutant models. Excess mortality was associated with particulate matter of aerodynamic diameter ≤10 μm/m^3^ (PM_10_), but not with other pollutants, in these two pollutant models. The extent of bias due to measurement error in these reported results is unclear. Schwartz and Coull recently proposed a method that deals with multiple exposures and, under certain conditions, is resistant to measurement error. We applied this method to reanalyze the data from NMMAPS. For PM_10_, we found results similar to those reported previously from NMMAPS (0.24% increase in deaths per 10-μg/m^3^ increase in PM_10_). In addition, we report an important effect of carbon monoxide that had not been observed previously.

Growing evidence from published studies has shown increased all-cause and specific-cause mortality from short-term exposures to air pollution (Fairley 1999; Katsouyanni et al. 1997; Pope at al. 1995; Schwartz 1993; Schwartz and Dockery 1992). An important piece of that evidence comes from the National Mortality and Morbidity Air Pollution Study (NMMAPS) conducted across 90 U.S. cities (Dominici et al. 2000a; Samet et al. 2000a, 2000b, 2000c). Recent updates of this study reported excess mortality in association with exposures to particulate matter of aerodynamic diameter ≤10 μm (PM_10_), whereas no independent associations with gaseous pollutants were observed (Dominici et al. 2002, 2003). In these previous studies, effects of pollutants were examined using two- and multiple-pollutant models.

From the public health perspective, when considering the evidence of a positive association between air pollution and mortality, it is important to determine whether such an effect is biased due to exposure misclassification and, if so, to correct for that bias.

The magnitude and direction of uncertainty in the observed effects of air pollution due to exposure measurement error have been argued by several investigators to be limitations in making causal inference for the link between air pollution and health outcomes (Lipfert and Wyzga 1997, 1999). In a single-pollutant model, exposure measurement error, due to the nondifferential misclassification, will underestimate the “true” effects of exposure–response associations (bias toward the null). Because of this, risk assessments based on the findings of observational epidemiologic studies may underestimate the benefits of reducing exposures. This is particularly true for air pollution studies, which, unlike cancer risk assessment, rely on maximum likelihood estimates of risk coefficients and not on upper confidence estimates.

The situation is more complex in the case of multiple correlated pollutants. Here, the measurement error in one pollutant will tend to bias the risk coefficient of that pollutant toward the null. However, measurement error in the second pollutant will contribute some bias to the coefficient of the first pollutant. The direction of the bias will depend on the sign of the correlation between the pollutants. In rare cases, when the correlation is high between the two pollutants and the measurement error in the second is large, this can produce an upward bias in the risk coefficient of the first pollutant. This may lead to an overestimation of exposure effects of the better measured pollutant (bias away from the null) (Schwartz 2000; Zeger et al. 2000). In the context of studies of air pollution, the upward bias has been cited as one reason for the positive associations between air pollutants and health outcomes (Lipfert and Wyuzga 1997, 1999). In recent analysis of this issue, Zeger et al. (2000) demonstrated that in the case of two pollutants measured with error, the correlation between the two pollutants, the variances of measurement errors of these two pollutants, and the correlation between the two errors would predict the magnitude and direction of bias. The study showed that even with hypothetically large differences in the four parameters, upward bias was unlikely (Zeger et al. 2000). Schwartz and Coull (2003) reported a similar finding in a simulation study, where under certain assumptions such as high correlation between the pollutants and their errors (> 0.95) and/or large difference in the error variances, upward bias was not likely to occur.

Why is there measurement error in air pollution studies? A recent development in air pollution epidemiology, called time-series design, is based on series of air pollution concentrations and health outcomes (events) over a certain period of observation (which may be months or years). Through this, one can estimate the average number of events that occur with changes in air pollution concentrations. The unit of analysis is the day, and the outcome data are the counts of events (mortality, or other health outcomes). Exposure data are usually ambient concentrations of different air pollutants measured continuously (hourly or daily) from fixed-site monitoring stations. However, monitored ambient concentrations of air pollutants are not representative of personal exposures, which are important when evaluating the relation of exposure and health outcome at the individual level. Unfortunately, in time-series studies of air pollution, there are no available calibration data (routinely measured series of personal exposure data) on which to base a measurement error correction. Dominici et al. (2000b) described a method to estimate a correction factor, using information on ambient and personal exposures in several cities in the United States, addressing only one pollutant (PM_10_). However, the applicability of that approach to the population-based time series is difficult because a more complex scenario of multiple air pollutants is generally present and usually information on only ambient exposures is available.

In recent reports, Schwartz and Coull (Schwartz 2000; Schwartz and Coull 2003) have developed an approach that uses hierarchical modeling to assess exposure–health outcome associations, which is resistant to exposure measurement error. The method is useful in studies with multiple exposures, such as air pollution time-series studies, and can provide bias-corrected estimates for the multiple exposures in the presence of measurement error. In the present study, we had two goals. First, to validate recent findings in air pollution epidemiology, we applied this approach to examine the independent effects of PM_10_ and several gaseous air pollutants on daily deaths, using recent data and results from NMMAPS. Second, we demonstrated the method with the intention to make it compelling to other researchers as a useful tool in assessing causal relationships.

## Materials and Methods

The NMMAPS study analyzed the association between air pollution and daily deaths in 90 U.S. cities. The cities included essentially the entire urban U.S. population living in counties with regular air pollution monitoring. Data on daily deaths in this study were obtained from records of the National Center for Health Statistics (Hyattsville, MD), and air monitoring data were obtained from the U.S. Environmental Protection Agency (Washington, DC), for the period from 1987 to 1994. This study conducted Poisson regressions, relating the daily death counts as a function of each day to air pollution concentrations on the same day, the previous day, and 2 days before the event, controlling for weather and season. Under such model, a Poisson process is assumed to describe the number of deaths per day, with events following a binomial process with low probability of occurrence. The model had the form log[*E*(*Y* )] = α+ ∑β*_j_X_j_* + ∑β*_k_Z_k_*, where *E* (*Y* ) was the expected daily death count, β*_j_* represented the coefficients measuring the effects of *j* pollutants, and β*_k_* represented the coefficients measuring the effects of *k* predictors (weather, season). The study looked at the effects on daily mortality from ambient concentrations of PM_10_, sulfur dioxide, nitrogen dioxide, carbon monoxide, and ozone. Further details have been published elsewhere (Dominici et al. 2003; Samet et al. 2000b, 2000c).

### The hierarchical model.

The approach from Schwartz and Coull (2003) applied to this study was as follows: If an outcome were linearly associated with two exposures—in our case two pollutants (*X*_1_ and *X*_2_)—then we would have a model such as


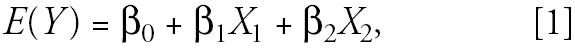


where *E* (*Y* ) is the expected daily mortality and β_1_ and β_2_ are the unbiased effects of, respectively, *X*_1_ and *X*_2_. If *X*_1_ and *X*_2_ were correlated with each other, then we could also fit a model like the following:


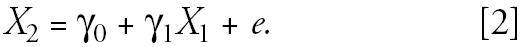


If we now substitute *X*_2_ in Equation 1 with Equation 2, then we would obtain


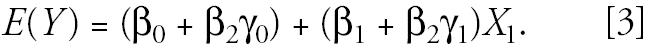


If we were to regress *Y* against *X*_1_ alone, then


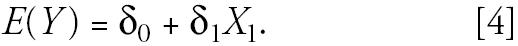


And by comparing Equations 3 and 4, we would obtain


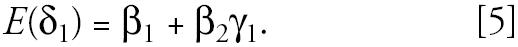


Hence, as Equation 5 shows, by regressing the coefficient relating *X*_1_ to mortality (δ_1_) against the coefficient relating *X*_2_ to *X*_1_ (γ_1_), we can recover β_2_, the coefficient relating *X*_2_ to mortality. If instead of substituting for *X*_2_ we had substituted for *X*_1_, then we would similarly have obtained an estimate of β_1_.

The advantage of this method is seen when we consider the impact of measurement error. If *X*_1_ and *X*_2_ are both measured with error, then the coefficients γ_1_ and δ_1_ in Equations 2 and 4 are both biased; however, that bias depends only on the variance of *X*_1_ and its measurement error, which are the same in both equations and cancel out in Equation 5. This results in the estimate of β _2_ in Equation 5 being unbiased. The extension of the approach to models with additional predictors, and Poisson regressions, is provided in Schwartz and Coull (2003).

We applied the hierarchical model to the NMMAPS study to estimate the unbiased independent effects of each of the five pollutants (PM_10_, SO_2_, NO_2_, CO, and O_3_) on daily mortality. One can think of the application of the hierarchical approach of Schwartz and Coull (2003) as a three-step analysis. As an example, we are presenting each step using two pollutants (e.g., SO_2_ and PM_10_). If we were to estimate the “true” effects of each of the two pollutants on daily mortality, then β_1_ (in Equation 1) would represent the unbiased effect of SO_2_, and β_2_ the unbiased effect of PM_10_. However, we are unable to estimate β_1_ and β_2_ directly, due to measurement error in each pollutant. The three-stage method then comes into play.

In the first stage, daily values of the various air pollutants, in each city, were regressed against each other to obtain regression slopes for each pollutant pair, using least-squares linear regression. In this step, we applied Equation 2, which in our example would take the form PM_10_ = γ_0_ + γ_1_SO_2_ + *e*. To enhance comparability with the original NMMAPS results, we obtained the daily air pollution data used by the NMMAPS researchers, so that their cleaning and averaging procedures would be reflected in our analysis. These data have been made publicly available as part of the Internet-based Health and Air Pollution Surveillance System (IHAPSS 2003).

The second stage involved fitting single-pollutant models to the mortality data, in each city, for each of the pollutants being examined (Equation 4). In such case, if estimating the effects of SO_2_ on daily mortality, then *E* (*Y* ) = δ_0_ + δ_1_SO_2_. The NMMAPS study already provided results for the single-pollutant models in each city, relating daily death counts to daily concentration of each pollutant (PM_10_, SO_2_, NO_2_, CO, and O_3_) on the day before (lag 1) the event, using Poisson regression modeling (Dominici et al. 2003; Samet et al. 2000b). Therefore, the slopes relating mortality and each of the air pollutants the day before death, by city, as provided by the NMMAPS, were the coefficients (δ_1_) of the second stage of the hierarchical approach.

Finally, the slopes (δ_1_) obtained from the second stage were regressed against those (γ_1_) obtained from the first stage, across the 90 U.S. cities (Equation 5), using least-squares linear regression. The slope of this final regression is, ideally, an unbiased estimate of β_2_, the effect of PM_10_ on daily mortality, controlling for the effect of SO_2_ and of measurement error.

The method allows one to recover the independent effect of each pollutant, controlling for any other pollutant, using an approach that is, in principle, unbiased by measurement error and that, in simulations, appears to be relatively unbiased under moderate violations of the model assumptions (Schwartz and Coull 2003). Obviously, for this approach to work, the independent variable (γ_1_) in Equation 5 must vary across the cities.

In the application of the three-stage analyses, the five pollutants were paired with each other. That is, for each pollutant, four unbiased independent estimates relating that pollutant to daily mortality were obtained, each estimate controlling for the effects of the other four pollutants. One limitation of the hierarchical modeling is loss in precision in these estimates, because the last regression (Equation 5) has only 90 observations. This is reflected in the confidence limits of the estimates. We could improve power in the effect of each pollutant, by averaging its four estimates, using the following formula:


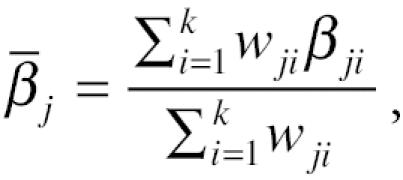


where β̄*_j_* is the weighted average slope (meta-slope) for pollutant *j* (i.e., PM_10_) defined over *k* number slopes (obtained from third-stage regressions), and β*_ji_* is the unbiased slope of pollutant *j* (i.e., PM_10_) controlling for pollutant *i* (i.e., SO_2_), where *i* = 1 to *k*. Weights for the slopes were defined as follows:


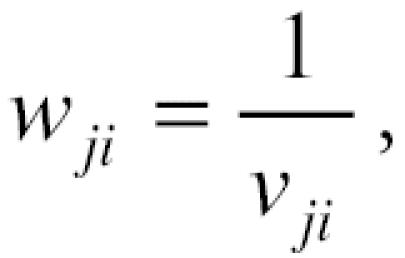


where *v_ji_* is the variance of β*_ji_*. The variance of β̄*_j_* is calculated as


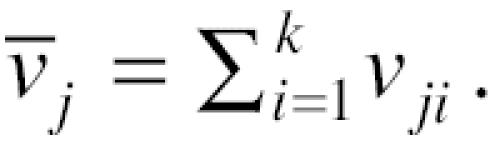


## Results

The relationship between pollutant pairs differed across the 90 cities (Table 1). The heterogeneity in the pollutant–pollutant regression slopes (γ_1_) among all the cities, as shown in Table 1, assured sufficient variability in the independent variable of the third-stage regression to proceed with the analysis. Power in a linear regression is increased by increasing the variability in the independent variable (γ_1_ in this case) and by reducing variability in the residuals. Table 1 indicates that at least the first condition is met. The range of variability in slopes relating PM_10_ to other pollutants was lower for traffic-related pollutants (CO and NO_2_) than for SO_2_ or O_3_. This likely reflects traffic particles always being a substantial component of PM_10_, whereas the correlation with SO_2_ is more varied because it depends on the sulfur content of fuel. SO_2_ is poorly correlated with O_3_, resulting in a very small mean slope and large range of variation. The range of variation in the slope relating NO_2_ to SO_2_ was smaller than for the other pollutants. It is possible that this reflects the importance of diesel emissions for NO_2_ concentrations in urban areas. Diesel fuel has much higher sulfur content than does gasoline, and this may contribute to a tighter spread of the association between the two pollutants across cities.

The bias-corrected estimates from the third-stage analysis are presented in Table 2. The results presented are percent increase in daily deaths for a 10-μg/m^3^ increase in PM_10_, or a 10-ppb increase in each of the gaseous air pollutants, except CO (100 ppb). For PM_10_, the percent increase in daily mortality ranged from 0.14 to 0.35% (controlling for other pollutants), with an overall estimate of 0.24% [95% confidence interval (CI), 0.05–0.42%]. In contrast, we found small and nonsignificant associations of daily deaths with SO_2_, NO_2_, and O_3_.

We found increased daily mortality in association with CO in the present analyses, with an overall relative excess daily mortality of 0.06% per increments of 100 ppb of CO, estimated with fair precision (95% CI, 0.02–0.10%).

## Discussion

The validity of exposure–response associations in epidemiologic studies depends on the precision of exposure measurements (Dominici et al. 2000b; Schwartz and Coull 2003; Zeger et al. 2000). Environmental studies of air pollution often lack precisely measured exposures, which can lead to exposure misclassification and biased estimates of exposure–response associations (Dominici et al. 2000b; Schwartz and Coull 2003; Zeger et al. 2000). Usually, an exposure measured with error will bias the association toward the null. A more complicated problem occurs when two or more exposures are measured with error and are correlated with each other. Zeger et al. (2000) reported that, in such a case, the better measured exposure may, rarely, capture some of the effect of the other exposure, which could bias away from the null. In any event, the extent of downward bias in each pollutant effect still depends on the measurement error in the other exposure. Hence, environmental studies of multiple air pollutants may inherit bias in both directions and with varying degrees. Underestimating the public health consequences of air pollution exposure can result in suboptimal measures to reduce these health consequences, particularly when cost–benefit or cost-effectiveness analysis is used as part of the decision process. Uncertainties about upward bias in the effect estimates can undermine the credibility of observed associations, raising questions about the appropriateness of proposed air quality standards. Hence, reducing both potential errors can improve environmental health.

Schwartz and Coull (2003) recently described a method that uses hierarchical modeling to deal with confounding and measurement error bias in epidemiologic studies. Their approach yielded an exposure–response estimate that was unbiased under certain assumptions and showed small downward biases when those assumptions were not met—for example, when the measurement errors among the multiple exposures were correlated. Although not entirely eliminated, the bias effect in this case was much less than the one produced by the two-pollutant model under the same circumstances (Schwartz and Coull 2003).

In the present study we applied the method of Schwartz and Coull (2003) to data and results from NMMAPS to estimate the independent effects of air pollutants on daily mortality. The application of this approach to the NMMAPS results was used in the case of two concurrent pollutants, both assumed to be measured with error. The method provided minimally biased independent effect estimates for each pollutant–daily mortality association. The price of this reduced bias was a reduction in precision. However, when we averaged over the results for each different pollutant, important associations appeared for PM_10_ and CO.

Recent results of NMMAPS had shown positive associations between PM_10_ and daily mortality for 90 U.S. cities (Dominici et al. 2003; Samet et al. 2000b, 2000c). The relative increase ± SE in daily mortality was 0.21 ± 0.06% per 10-μg/m^3^ increase of PM_10_ concentration 1 day before the event. The presence of other pollutants in the model did not change this effect (Dominici et al. 2003). Our estimate for this pollutant was slightly greater after reducing measurement error bias (0.24 ± 0.09% increase in mortality).

The NMMAPS had reported no independent associations between daily mortality and concentration of other air pollutants 1 day before the event, including SO_2_, NO_2_, CO, and O_3_ (Dominici et al. 2003). The findings for gaseous pollutants from that study were based on two- and multiple-pollutant models. For comparison with our results, we report the estimates from this previous study for increments in concentration of 10 ppb. The estimates for SO_2_ from the two- and multiple-pollutant models from the NMMAPS ranged between 0.4 and 0.5% increase in daily mortality. NO_2_ showed relative increases in daily mortality from 0.3 to about 0.4%. Relative increase in daily mortality for O_3_ varied between 0.08 and 0.2%. Percent increases of 0.02–0.06 in daily mortality were associated with increments of 100 ppb in CO concentrations. None of these reported effects was estimated precisely, which resulted in the summary of the evidence from this previous study of no association between any of the gaseous pollutants and daily mortality (Dominici et al. 2003).

We found the effects for SO_2_, controlling for other pollutants, to vary greatly, with an overall estimate of 0.1% increase in daily mortality. For NO_2_, we found essentially no effect on daily mortality (estimate = –0.004%). O_3_ effects in our study were found to be between two and three orders of magnitude smaller than the observed effects for the same pollutant from the NMMAPS. However, none of these estimates was precise, which made our summary finding for these pollutants qualitatively similar to the one reported from the NMMAPS study.

Unlike in the NMMAPS finding, we observed an association between CO and daily mortality. The estimates, controlling for other pollutants, ranged from −0.02 to 0.09%, with the greater effects being estimated fairly precise. The pooled effect of CO showed a 0.06% increase in mortality with a tight confidence interval (95% CI, 0.02–0.10% per 100 ppb).

One explanation for the different finding for CO in our study, compared with that of NMMAPS, could be related to the high degree of measurement error in this pollutant. There is a possibility that the spatial heterogeneity in ambient concentrations of CO is greater than that of any other air pollutant. This would produce a greater amount of measurement error when monitoring ambient concentrations of CO from a central monitoring site. Whether the greater effect seen using the hierarchical modeling approach reflects a true association with CO per se, or whether CO is a surrogate for traffic particles or some other component of vehicular exhaust (Sarnat et al. 2001), is not clear. Nevertheless, the results for CO indicate the potential use of the approach and suggest that attention should be focused on CO or on traffic pollution. The similar results of this study with those of NMMAPS for PM_10_ were reassuring.

Several limitations in the application of the Schwartz and Coull (2003) approach must be acknowledged. First, because the third-stage regression had only 90 observations, the power of the method was reduced. The power depends in part on the *R* ^2^ values of the third-stage models. In our case, these were low (Table 2), forcing us to apply meta-analysis as an ad hoc approach to improve power. In other applications, *R*^2^ may be larger, and this approach may be unnecessary. We are currently developing a multivariate version of the approach that is less ad hoc in improving power, by using multiple predictors at the last-stage regression.

Second, we did not have season-specific regression coefficients relating air pollution to mortality in the NMMAPS. The use of this approach with season-specific relationships offers increased power because of the increased number of risk coefficients by pollutant, and also because the relationship between many pollutants varies seasonally. We expect these to be available from NMMAPS in the future.

Third, in the present analyses we assumed that the concentration–response relation between air pollution and daily deaths is linear. This question has been subject to a number of investigations using splines and smoothing in single-city studies and combining splines or smooth curves in multicity studies (Schwartz and Zanobetti 2000; Schwartz et al. 2002). At least for PM_10_, linear associations were seen in concentration ranges similar to those of the present study.

Finally, our model implies that each pollutant, as measured at a central site in each city, is a surrogate for exposure to the same pollutant. However, Sarnat et al. (2001) have proposed recently that ambient concentrations of gaseous air pollutants may be serving as surrogates not for exposure to those gases but for exposure to particles or particles from particular sources. For example, in Baltimore, CO was a good surrogate for exposure to particles from traffic (Sarnat et al. 2001). This suggests that there must be caution in interpreting the present results for CO. We think that personal exposure studies of intermediate outcomes may be necessary to resolve the question.

Despite these limitations, the approach of Schwartz and Coull (2003) is useful to studies of air pollution and mortality. In our study, it provided evidence of a slightly greater effect of PM_10_ than that reported previously by NMMAPS, when controlling for measurement error in other pollutants, and suggested that greater attention be paid to CO and possibly traffic pollution.

## Figures and Tables

**Table 1:** Mean ± SD of the distribution of pollutant–pollutant regression slopes (γ_1_),*^a^* and their coefficients of variation (CV), across 90 U.S. cities, for each pollutant pair.

Pollutant–pollutant regression variables		Pollutant–pollutant regression slopes	
Dependent pollutant	Independent pollutant	Mean ± SD	CV (%)
PM_10_	SO_2_	1.31 ± 1.45	110.3
	NO_2_	0.83 ± 0.64	77.2
	CO	0.01 ± 0.01	62.7
	O_3_	0.27 ± 0.38	143.6
SO_2_	PM_10_	0.09 ± 0.08	87.9
	NO_2_	0.22 ± 0.20	91.6
	CO	0.003 ± 0.003	92.0
	O_3_	−0.03 ± 0.10	377.6
NO_2_	SO_2_	2.64 ± 5.09	192.8
	PM_10_	−0.22 ± 1.83	831.1
	CO	−0.01 ± 0.08	1030.7
	O_3_	0.19 ± 2.03	1064.6
CO	SO_2_	61.58 ± 74.64	121.2
	NO_2_	34.19 ± 19.39	56.7
	PM_10_	13.73 ± 9.26	67.4
	O_3_	−9.58 ± 9.47	98.8
O_3_	SO_2_	136.66 ± 535.82	392.1
	NO_2_	24.76 ± 95.82	387.0
	CO	−0.59 ± 3.26	550.6
	PM_10_	−63.99 ± 292.61	457.2

a The slopes were obtained from first-stage regressions (Equation 2), pairing each of the five pollutants with the other four (pollutant pairs), for each U.S. city.

**Table 2:** Percent increase in daily deaths associated with each pollutant, controlling for measurement error in other pollutants, based on data and results from the NMMAPS study, including the period between 1987 and 1994.

	Independent effects of pollutants on daily mortality*^a^*														
	PM_10_			SO_2_			NO_2_			CO			O_3_		
Paired pollutant*^b^*	*R*^2^*^c^*	Slope*^d^*	*T**^e^*	*R*^2^	Slope	*T*	*R*^2^	Slope	*T*	*R*^2^	Slope	*T*	*R*^2^	Slope	*T*
PM_10_	NA	NA	NA	0.048	1.14	1.77	0.008	0.033	0.69	0.029	0.078	1.56	0.008	0.0003	0.77
SO_2_	0.047	0.28	1.73	NA	NA	NA	< 0.001	−0.004	0.06	0.154	0.086	3.25	0.012	−0.0004	0.83
NO_2_	0.012	0.16	0.83	0.007	−0.29	0.54	NA	NA	NA	0.007	0.032	0.64	0.024	−0.0019	1.15
CO	0.006	0.14	0.69	0.030	0.65	1.33	0.001	−0.004	0.27	NA	NA	NA	0.045	0.0011	1.87
O_3_	0.036	0.35	1.70	0.039	−0.76	1.52	0.007	−0.025	0.63	0.002	−0.018	0.34	NA	NA	NA
Meta-slope*^f^*	NA	0.24	2.80	NA	0.10	0.34	NA	−0.004	0.27	NA	0.062	3.16	NA	0.0002	0.74

NA, nonapplicable.

a Corrected slopes (effects) removing the effect of each pollutant shown in the first column.

b The independent pollutant of first-stage regression.

c Adjusted *R*^2^ from regressions of the third stage (see text for details).

d Slope for PM_10_ presented as percent increase per 10 μg/m^3^. Slopes for other pollutants are presented as percent increase per 10 ppb (100 ppb for CO).

e *t*-Statistic from regressions of the third stage.

f Meta-slope of the four alternative estimates.
